# PReS13-SPK-1253: Challenges in nursing care with the new biologics for JIA

**DOI:** 10.1186/1546-0096-11-S2-I2

**Published:** 2013-12-05

**Authors:** P Livermore

**Affiliations:** 1 Rheumatology, Great Ormond Street, London, UK

## 

Sam was diagnosed with extended oligoarticular Juvenile Idiopathic Arthritis at the age of 18 months. He began Etanercept at the age of 10 years old in March 2007 after an inadequate response to non-steroidal medications, methotrexate and numerous steroid preparations. Seventeen months later he was switched to Adalimumab with the expectation that this would have an improved response for him. Sam hated Adalimumab and was struggling to tolerate the injections. Encouraging Sam to persevere was difficult but he did so. After seven months, Sam refused anymore Adalimumab and had fourteen months only on Methotrexate, with numerous joint injections and intravenous steroid pulses. He was then commenced on Abatacept. Sam was seen every 4 weeks and his active joint disease remained apparent. By this time Sam had stopped playing football, socialising with his friends and was feeling frustrated with the limitations his arthritis posed on his daily life.

After eleven months Sam was offered his 4^th ^Biological therapy - Tocilizumab. Whilst Sam has not got systemic disease, the options available were fast decreasing. His family were encouraging about a new therapy that like the others "might just be the one for him". Sam on the other hand was reluctant and needed persuasion. After some response, the decision was made to increase the dose past the recommended dose. This medication like the others had challenges with it for Sam; such as rashes, low neutrophils, and when he became unwell at home, this was not escalated appropriately as his ESR and CRP were normal in the absence of fever. There have also been issues for the health professionals in his care; applying to begin the medication, needing a fortnightly day-case bed, abnormal blood parameters and the concern about how to control Sam's disease. Still requiring frequent joint injections and occasional intravenous pulses of steroids when he allows it, Sam is getting very fed up with the fortnightly infusion and the associated day out of school. Now at 16 years of age and struggling with his GCSE exams, Sam wants to know what will work for him. Despite his parent's strong reluctance, Sam wants to know if he can have a bone marrow transplant and believes the quoted 10% risk of mortality is worth it.

Sam's case is not unique but it does highlight many issues with these newer 'biological' therapies. This presentation will highlight many of these issues including injection site reactions, adverse events, changing therapies and perhaps most importantly; managing patient expectations.

## Disclosure of interest

None declared.

